# Supplementary material for: Comparative multiomics analysis of cell physiological state after culture in a basket bioreactor
Source: Sci Rep. 2022 Nov 23;12:20161. doi: 10.1038/s41598-022-24687-4 (PMC9686226; doi:10.1038/s41598-022-24687-4)

23666038.1:83144994:83145374:-@NW_023666038.1:83151640:83152298:-@NW_023666038.1:83152140:8315[illegible]

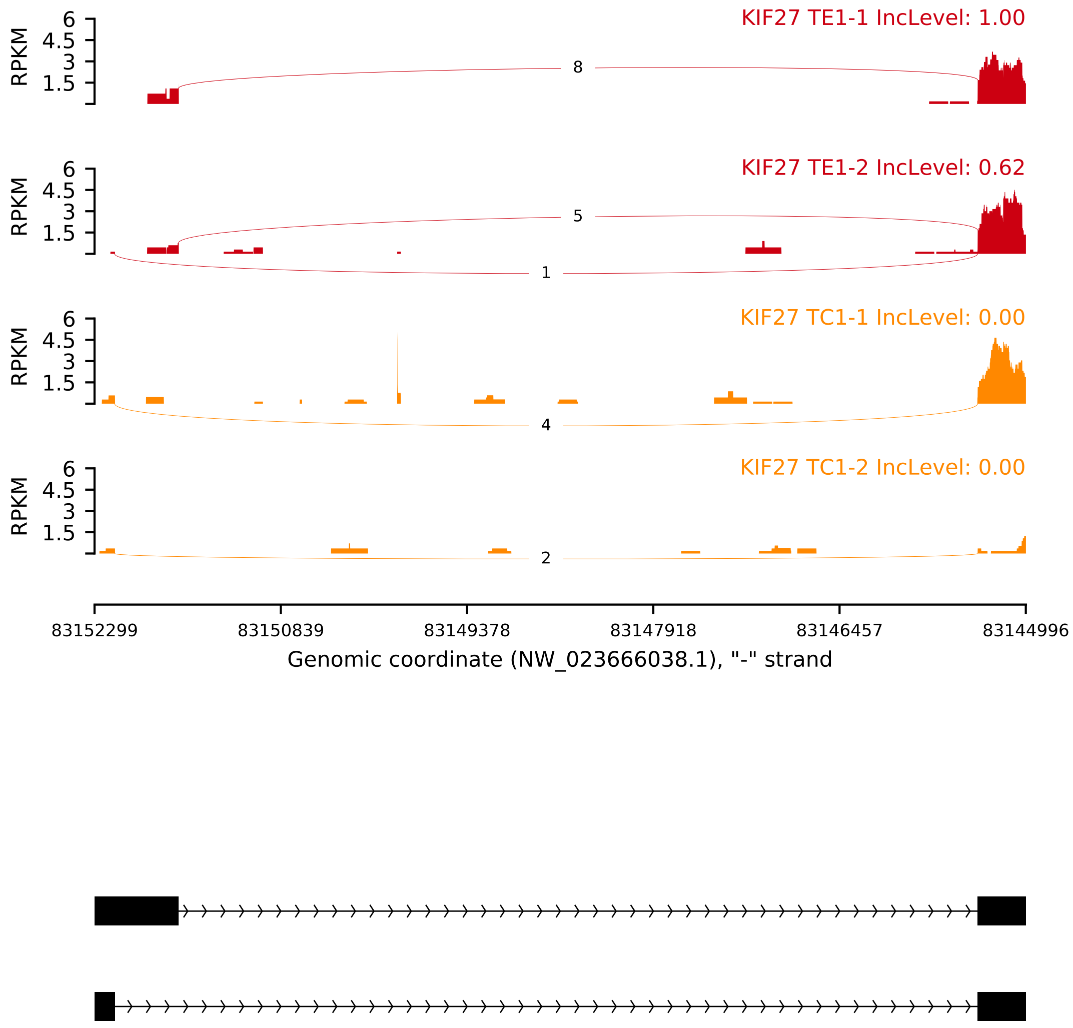

Supplement: Supplementary file 1 — Supplementary Information 1. [file 41598_2022_24687_MOESM1_ESM.zip › raw data/Transcriptomic raw data/7.AS/2.dasplot/TE1vsTC1/A5SS/16_KIF27_NW_023666038.1_83144994_83145374_-@NW_023666038.1_83151640_83152298_-@NW_023666038.1_83152140_83152298_-.pdf]
